# Electroconvulsive Therapy and the Risk of Suicide in Hospitalized Patients With Major Depressive Disorder

**DOI:** 10.1001/jamanetworkopen.2021.16589

**Published:** 2021-07-21

**Authors:** Ida Rönnqvist, Fredrik K. Nilsson, Axel Nordenskjöld

**Affiliations:** 1 School of Medical Sciences, Örebro University, Örebro, Sweden; 2 University Health Care Research Centre, Faculty of Medicine and Health, Örebro University, Örebro, Sweden

## Abstract

**Importance:**

Electroconvulsive therapy (ECT) is indicated for severe depression, including depression with psychosis, catatonia, and/or an elevated suicide risk. However, the association of ECT with suicide risk is uncertain.

**Objective:**

To determine the association between ECT and the risk of suicide in patients with unipolar major depressive disorder.

**Design, Setting, and Participants:**

This registry-based cohort study used patient data from Swedish national registers. Patients with a record of inpatient care between January 1, 2012, and October 31, 2018, for moderate depression, severe depression, or severe depression with psychosis were included in the study. Propensity score matching (1:1) was used to balance risk factors for suicide at baseline between patients treated with and without ECT during the inpatient episode.

**Exposures:**

Data from the Swedish National Quality Register for ECT and the Swedish National Inpatient Register were combined to identify patients who had received ECT during the inpatient episode. National registers were used to identify risk factors for suicide.

**Main Outcomes and Measures:**

Suicide within 3 and 12 months of admission to inpatient care was analyzed. Cox regression analyses were used to adjust for confounders.

**Results:**

The study included 28 557 patients (mean [SD] age, ECT group, 55.9 [18.4] years; non-ECT group, 45.2 [19.2] years; 15 856 women [55.5%]). In the matched sample of 5525 patients in each group, 62 patients (1.1%) in the ECT group and 90 patients (1.6%) in the non-ECT group died of suicide within 12 months (hazard ratio [HR], 0.72; 95% CI, 0.52-0.99). Electroconvulsive therapy was significantly associated with a decreased risk of suicide in patients with psychotic features (HR, 0.20; 95% CI, 0.08-0.54) and those aged 45 to 64 years (HR, 0.54; 95% CI, 0.30-0.99) or 65 years or older (HR, 0.30; 95% CI, 0.15-0.59), but not in patients aged 44 years or younger (HR, 1.22; 95% CI, 0.68-2.16).

**Conclusions and Relevance:**

The results of this cohort study support the continued use of ECT to reduce suicide risk in hospitalized patients who are severely depressed, especially those who are older than 45 years and those with a psychotic subtype.

## Key Points

**Question:**

What is the association of electroconvulsive therapy (ECT) with the risk of suicide among hospitalized patients diagnosed with major depressive disorder?

**Findings:**

In this registry-based cohort study of 28 557 hospitalized patients, ECT was associated with a significantly reduced risk of suicide in patients with major depressive disorder. In the matched sample of 5525 patients in each group, 62 patients (1.1%) in the ECT group and 90 patients (1.6%) in the non-ECT group died of suicide within 12 months.

**Meaning:**

The results of this cohort study support the continued use of ECT to reduce suicide risk among hospitalized patients who are severely depressed, especially older patients and those with psychotic features.

## Teaser

This cohort study uses data from the Swedish National Quality Register to investigate the association of electroconvulsive therapy with suicide risk in hospitalized patients diagnosed with major depressive disorder.

## Introduction

In patients with depression, the risk of suicide is elevated for many years after discharge from psychiatric inpatient care and is highest during the first 3 months after discharge.^1^ Electroconvulsive therapy (ECT) is an effective treatment for depression^2^ that, according to the guidelines of the American Psychiatric Association,^3^ is indicated for patients with severe major depression, including those with psychosis, catatonia, and/or an elevated suicide risk.^3^ However, the use of ECT is still debated, in part because there is a lack of data regarding the beneficial effects on suicide risk.^4^

Given that ECT is an effective treatment for depression, especially for older patients and those with psychotic symptoms,^5^ it is expected to reduce the risk of suicide. Although some data have revealed ECT-associated reductions in suicidal ideation^6^ and suicide attempts,^7^ to our knowledge, few studies have investigated the association of ECT with suicide rate. A recent large Danish register-based study showed that ECT is associated with an increased risk of suicide compared with non-ECT.^8^ Other smaller studies reported that ECT is associated with both an increased^9^ and decreased^10^ suicide risk. These conflicting results, which could be explained by incomplete adjustment for potential confounding factors, mean that the effect of ECT on suicide risk is uncertain. In the current study, we used more detailed population registers than previous studies to examine the association between ECT indicated for depression and the risk of suicide. We hypothesized that ECT was associated with a decreased suicide risk, especially among older patients and patients with more severe forms of depression.

## Methods

### Study Design and Participants

This cohort study used data obtained from Swedish national registers and was reported according to the Strengthening the Reporting of Observational Studies in Epidemiology (STROBE) reporting guideline. Patients who had a record of inpatient care for moderate depression as indicated by the *International Statistical Classification of Diseases and Related Health Problems, Tenth Revision (ICD-10)* codes F32.1, F33.1, severe depression (F32.2, F33.2), or severe depression with psychosis (F32.3, F33.3) between January 1, 2012, and October 31, 2018, were included in the study. Electroconvulsive therapy is not indicated as a treatment for mild depression, and patients with mild depression or unspecified depression were therefore excluded. Patients with a history of manic episodes, bipolar disorder, schizophrenia, schizotypal disorders, or delusional disorders were also excluded. Individuals younger than 18 years were not included in the study. Patients who had received ECT during the inpatient episode comprised the ECT group. The non-ECT group comprised patients who had not been treated with ECT during the inpatient period. The study was approved by the Regional Ethical Review Board in Uppsala, Sweden (registration No. 2014/174), and the need for informed consent was waived because patients were not identifiable.

### Procedures

The outcome of interest was suicide. Suicide was defined as death caused by intentional self-harm (*ICD-10* codes X60-X80) or by an event of undetermined intent (*ICD-10* codes Y10-Y35). Risk factors for suicide in individuals with depression that have been identified by previous studies were included as covariates. These factors included older age,^11^ male sex,^12^ a greater severity of depression, a family history of mental disorder,^12^ and previous suicide attempts or self-harming behavior.^12^ Similar risk factors have been reported for suicide in the general population.^13^ Psychiatric comorbidities (anxiety and alcohol and drug use disorders),^12^ somatic comorbidities (chronic obstructive pulmonary disease, cancer, spinal disease, asthma, stroke, diabetes, and ischemic heart disease),^13^ unemployment,^13,14,15^ living alone,^15^ and a family history of suicide^16^ are other risk factors for suicide in the general population and were also included as covariates in the present study. We evaluated the risk factor of the recent death of a child, but we did not identify any deaths within 6 months of the end of follow-up and did not include this factor.^17^ The association between education level and suicide varies; some studies identified no relationship,^18^ whereas others reported that both low^13,19^ and high^20^ education levels increase the risk of suicide. A high level of parental education has been reported to be a protective factor against suicide.^21^ The education level of both the patients and their parents were therefore included in the study. Treatment with lithium reduces the risk of suicide for patients with mood disorders.^22^ Several studies have also reported that antidepressant treatment is associated with a decreased risk of suicide.^23,24^ Use of these medications in the 100 days before the inpatient episode was therefore included in the analyses. The follow-up periods analyzed were 3 and 12 months from the date of admission to inpatient care. To control for pharmacologic treatment during follow-up, additional sensitivity analyses were performed including use of lithium and antidepressants.

### Data Sources

The Swedish National Inpatient Register (IPR) is a mandatory register of inpatient care with a coverage of more than 99%,^25^ from which the patients hospitalized for depression were identified (diagnoses based on *ICD-10*). The use of ECT was identified by corresponding IPR intervention codes. Data on age, sex, admission and discharge dates, secondary diagnoses, and previous self-harm were also obtained from the IPR. The Swedish National Quality Register for ECT (Q-ECT) is a national register established in 2011 that contains information about ECT in Sweden.^26^ We combined data from the Q-ECT and IPR to identify subjects who had received ECT. Data were also collected from the Swedish Prescribed Drug Register,^27^ Swedish Causes of Death Register,^28^ and Longitudinal Integration Database for Health Insurance and Labour Market Studies, which contain demographic and socioeconomic data of all Swedish citizens older than 15 years.^29^ The Multi Generation Register was used to gather information about family histories of mental disorders and suicide. Details regarding the diagnoses and procedural codes used are available in eTables 1-3 in the Supplement.

### ECT Use

Electroconvulsive therapy was usually administered 3 times per week using the bidirectional constant-current brief-pulse Mecta (Mecta Corp) or Thymatron (Somatics Inc) device. The electrode application during ECT was unilateral for 4781 of 5525 patients (86.5%), bilateral for 539 patients (9.8%), and not known (data missing) for the remaining 205 patients (3.7%). Further details regarding ECT are available in eTable 4 in the Supplement.

### Statistical Analyses

Baseline characteristics were compared between the ECT and non-ECT groups using standardized mean differences (Table 1). We matched ECT and non-ECT groups exactly in terms of sex, age group, and severity of depression based on diagnosis codes. We further matched the groups by using the propensity score of the patients receiving ECT. The propensity score was calculated by a logistic regression model using age, marital status, living alone, employment status, education level, parental education level, family history of mental disorder, family history of suicide, compulsory psychiatric treatment, anxiety disorder, personality disorder, alcohol use disorder, substance use disorder, diabetes, obstructive airway disease, ischemic heart disease, heart failure, stroke, cancer, spinal disease, suicide attempt by poisoning, suicide attempt by another method, antidepressant use, and lithium use as factors. A univariate Cox regression model with the treatment variable was used to compare the suicide risk between the 2 treatment groups within 3 and 12 months of the first inpatient episode during the study period in the matched sample. Cases were censored if non–suicide-related death occurred or follow-up ended. To increase statistical power, we used all inpatient episodes in the study period in the total cohort in a Cox regression model with 3 months and 12 months of follow-up to identify subgroups with differential suicide risks between treatment groups. In the episode-based analysis, we attributed each suicide to the latest inpatient episode if it occurred within 3 or 12 months of admission to inpatient care. Interaction analysis and stratified analyses were performed (Table 2). The risks are expressed as hazard ratios (HRs) with 95% CIs. All *P* values were 2-sided, and significance was defined as *P* < .05. A Kaplan-Meier graph illustrates the suicide risk in the matched sample. There were 19 patients (0.1%) with missing demographic information from the Longitudinal Integration Database for Health Insurance and Labour Market Studies; these patients were imputed into the largest category. Data management and statistical analyses were performed using SAS, version 6.1 (SAS Institute) and SPSS, version 22.0 (SPSS Inc).

**Table 1.** Baseline Characteristics of Patients With Depression Treated With and Without Electroconvulsive Therapy Before and After Matching

Variable	Group, No. (%)		SMD	Matched group, No. (%)		SMD
	ECT (n = 6412)	Non-ECT (n = 22 145)		ECT (n = 5525)	Non-ECT (n = 5525)	
**Sex**						
Male	2727 (42.5)	9974 (45.0)	−0.05	2321 (42.0)	2321 (42.0)	0.00
Female	3685 (57.7)	12 171 (55.0)		3204 (58.0)	3204 (58.0)	
Age, y						
18-24	352 (5.5)	3845 (17.4)	−0.56	316 (5.7)	316 (5.7)	0.00
25-34	711 (11.1)	4171 (18.8)		670 (12.1)	670 (12.1)	
35-44	785 (12.2)	3374 (15.2)		739 (13.4)	739 (13.4)	
45-54	1025 (16.0)	3765 (17.0)		986 (17.8)	986 (17.8)	
55-64	1096 (17.1)	2783 (12.6)		943 (17.1)	943 (17.1)	
65-74	1278 (19.9)	2156 (9.7)		1011 (18.3)	1011 (18.3)	
≥75	1165 (18.2)	2051 (9.3)		860 (15.6)	860 (15.6)	
Severity of depression						
Moderate	1239 (19.3)	11 159 (50.4)	−0.65	1174 (21.2)	1174 (21.2)	0.00
Severe without psychosis	3440 (53.6)	8045 (36.3)		3037 (55.0)	3037 (55.0)	
Severe with psychosis	1733 (27.0)	2941 (13.3)		1314 (23.8)	1314 (23.8)	
Marital status						
Married or cohabiting	2564 (40.0)	5761 (26.0)	−0.30	2069 (37.4)	2125 (38.5)	0.02
Divorced	1153 (18.0)	3931 (17.8)	−0.01	1035 (18.7)	994 (18.0)	−0.02
Widowed	571 (8.9)	1206 (5.4)	−0.14	474 (8.6)	471 (8.5)	−0.00
Unmarried or unknown	2124 (33.1)	11 247 (50.8)	0.36	1947 (35.2)	1935 (35.0)	−0.00
Household						
Not living alone	3498 (54.6)	11 210 (50.6)	0.08	2929 (53.3)	2964 (53.6)	−0.01
Education level						
Low (≤9 y) or unknown	1619 (25.2)	6303 (28.5)	−0.11	1381 (25.0)	1342 (24.3)	0.01
Middle (10-12 y)	2855 (44.5)	10 181 (46.0)		2479 (44.9)	2530 (45.8)	
High (>12 y)	1938 (30.2)	5661 (25.6)		1665 (30.1)	1653 (29.9)	
Parental education level						
Low (≤9 y)	1383 (21.6)	3743 (16.9)	−0.12	1154 (20.9)	1144 (20.7)	−0.00
Middle (10-12 y)	1577 (24.6)	7072 (31.9)	0.16	1424 (25.8)	1446 (26.2)	0.01
High (>12 y)	1015 (15.8)	4958 (22.4)	0.17	950 (17.2)	951 (17.2)	0.00
Unknown	2437 (38.0)	6372 (28.8)	−0.20	1997 (36.1)	1984 (35.9)	−0.00
Employment status						
Employed	3374 (52.6)	13 214 (59.7)	0.14	3012 (54.5)	3034 (54.9)	0.01
Psychiatric comorbidity						
Anxiety disorder	1961 (30.6)	5976 (27.0)	−0.08	1659 (30.0)	1587 (28.7)	−0.02
Personality disorder	376 (5.9)	1184 (5.3)	−0.02	321 (5.8)	323 (5.8)	0.00
Alcohol use disorder	479 (7.5)	2531 (11.4)	0.13	442 (8.0)	441 (8.0)	0.00
Substance use disorder	492 (7.7)	2128 (9.6)	0.07	426 (7.7)	410 (7.4)	−0.01
Somatic comorbidity						
Drugs used for diabetes	509 (7.9)	1502 (6.8)	−0.04	429 (7.8)	424 (7.7)	−0.03
Drugs for obstructive airway diseases	1387 (21.6)	5347 (24.1)	0.06	1248 (22.6)	1187 (21.5)	−0.00
Ischemic heart disease	344 (5.4)	1069 (4.8)	−0.02	592 (5.4)	296 (5.4)	0.00
Heart failure	121 (1.9)	556 (2.5)	0.04	101 (1.8)	116 (2.1)	0.02
Stroke	198 (3.1)	783 (3.5)	0.02	174 (3.1)	178 (3.2)	0.01
Cancer	396 (6.2)	950 (4.3)	−0.09	312 (5.6)	307 (5.6)	0.00
Spinal disease	388 (6.1)	1401 (6.3)	0.01	345 (6.2)	321 (5.8)	−0.01
Suicide attempt—poisoning						
Within 1 y	417 (6.5)	1767 (8.0)	0.06	368 (6.7)	361 (6.5)	−0.00
More than 1 y ago	464 (7.2)	1755 (7.9)	0.03	422 (7.6)	390 (7.1)	−0.02
No	5531 (86.3)	18 623 (84.1)	−0.06	4735 (85.7)	4774 (86.4)	0.01
Suicide attempt—other method						
Within 1 y	95 (1.5)	323 (1.5)	−0.02	74 (1.3)	78 (1.4)	0.01
More than 1 y ago	144 (2.2)	412 (1.9)	−0.03	110 (2.0)	95 (1.7)	−0.02
No	6173 (96.3)	21 410 (96.7)	0.02	5341 (96.7)	5352 (96.9)	0.01
Pharmacotherapy						
Antidepressants	5842 (91.1)	18 216 (82.3)	−0.31	5012 (90.7)	5051 (91.4)	0.02
Lithium	492 (7.7)	838 (3.8)	−0.17	366 (6.6)	345 (6.2)	−0.01
Compulsory psychiatric treatment						
Yes	1126 (17.6)	3029 (13.7)	−0.11	950 (17.0)	915 (16.6)	−0.01
Family history of first degree						
Mental disorder	692 (10.8)	2518 (11.4)	0.02	596 (10.8)	603 (10.9)	0.00
Suicide	238 (3.7)	733 (3.3)	−0.02	204 (3.7)	194 (3.5)	−0.01

Abbreviations: ECT, electroconvulsive therapy; SMD, standardized mean difference.

**Table 2.** Association Between Treatment With Electroconvulsive Therapy and Suicide Risk in an Adjusted Model Stratified by Age Group and Severity of Depression^a^

Variable	aHR (95% CI)	*P* value	*P* value for interaction analyses	Total suicide events
Age groups, y				
18-44	1.22 (0.68-2.16)	.51	Reference	75
45-64	0.54 (0.30-0.99)	.05	.04^b^	76
≥65	0.30 (0.15-0.59)	.001	.001^b^	59
Severity of depression				
Moderate (F32.1, F33.1)^c^	0.93 (0.46-1.87)	.84	Reference	75
Severe without psychosis (F32.2, F33.2)	0.65 (0.41-1.04)	.07	.42^d^	99
Severe with psychosis (F32.3, F33.3)	0.20 (0.08-0.54)	.001	.01^d^	36

Abbreviation: aHR, adjusted hazard ratio.

^a^ Cox regression model during 90 days of follow-up after date of admission, stratified by age group and severity of depression. Interactions analyses are reported in a separate column.

^b^ *P* values for interaction with 18-44 y.

^c^ Numbers represent *International Statistical Classification of Diseases and Related Health Problems, Tenth Revision* codes.

^d^ *P* values for interaction with moderate severity of depression.

## Results

### Comparison of the ECT and Non-ECT Groups

The study population consisted of 28 557 patients (mean [SD] age, ECT group, 55.9 [18.4] years; non-ECT group, 45.2 [19.2] years; 12 701 men [44.5%] and 15 856 women [55.5%]). A total of 6412 patients (22.5%) were treated with ECT during the first inpatient episode in the study period. The total number of inpatient episodes was 43 169, of which 11 578 (26.8%) involved ECT; a comparison based on inpatient episodes is provided in eTable 5 in the Supplement. Of the study population, 5525 patients with a first episode of inpatient care during the study period in each group were successfully matched. Table 2 shows the baseline characteristics of the ECT and non-ECT groups before and after matching.

### Suicide Within 12 Months

In the matched sample, ECT was associated with a significantly decreased risk of suicide within 12 months compared with non-ECT (HR, 0.72; 95% CI, 0.52-0.99) (Figure). A total of 62 of 5525 patients (1.1%) in the ECT group and 90 of 5525 patients (1.6%) in the non-ECT group died of suicide within 12 months. In the episode-based analysis at 12 months, suicide risk did not significantly differ between the ECT and non-ECT groups in the main analysis (HR, 0.82; 95% CI, 0.65-1.03) or after adjustment for treatment with lithium and/or antidepressants during follow-up (HR, 0.93; 95% CI, 0.73-1.17).

**Figure.**
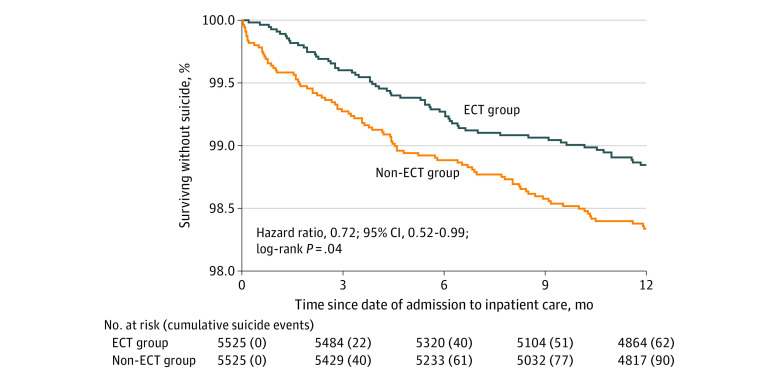
Kaplan-Meier Estimates of the Proportion of Patients Surviving Without Suicide Within 12 Months of Discharge After Inpatient Care for Depression With or Without Electroconvulsive Therapy (ECT)

### Suicide Within 3 Months

In the matched sample, there were 22 of 5525 (0.4%) suicides in the ECT group and 40 of 5525 (0.7%) suicides in the non-ECT group within 3 months, but this difference was not significant (HR, 0.66; 95% CI, 0.40-1.11). In the whole cohort, 210 of 28 557 patients (0.7%) committed suicide within 3 months; this included 41 of 6412 patients (0.6%) and 41 of 11 578 inpatient episodes (0.4%) in the ECT group and 169 of 22 145 patients (0.8%) and 169 of 31 591 inpatient episodes (0.5%) in the non-ECT group. In the episode-based analysis of the whole cohort with 3 months of follow-up, ECT was associated with a significantly lower risk of suicide than non-ECT (HR, 0.58; 95% CI, 0.40-0.83), and this association was retained in sensitivity analyses adjusting for treatment with lithium and/or antidepressants during follow-up (HR, 0.60; 95% CI, 0.42-0.86). The model is available in eTable 6 in the Supplement.

There was a significant interaction effect on suicide between the age group and treatment groups as well as between the treatment group and depression severity (Table 2). Electroconvulsive therapy was associated with a significantly lower risk of suicide than non-ECT among patients aged 45 to 64 years (HR, 0.54; 95% CI, 0.30-0.99) and those aged 65 years or older (HR, 0.30; 95% CI, 0.15-0.59). There was no statistically significant association between ECT and suicide among patients aged 18 to 44 years (HR, 1.22; 95% CI, 0.68-2.16). There was a significant association between reduced suicide risk and ECT in the stratified analysis of patients with severe depression and psychosis (HR, 0.20; 95% CI, 0.08-0.54; *P* = .001), but not of patients with moderate depression (HR, 0.93; 95% CI, 0.46-1.87) or severe depression without psychosis (HR, 0.65; 95% CI, 0.41-1.04) (Table 2).

### Mortality

In the matched sample, all-cause mortality was significantly lower in the ECT group than in the non-ECT group within 3 months (HR, 0.43; 95% CI, 0.30-0.61) and 12 months (HR, 0.67; 95% CI, 0.55-0.82). The numbers of deaths were 41 of 5525 (0.7%) at 3 months and 161 (2.9%) at 12 months in the ECT group compared with 96 (1.7%) at 3 months and 237 (4.3%) at 12 months in the non-ECT group (both comparisons, *P* < .001).

## Discussion

To our knowledge, few studies have adequately investigated the association between suicide risk and ECT, and their results are inconsistent. To clarify this issue, we performed a large-scale study that included extensive adjustment for confounders. We found that ECT for depression was associated with a reduced risk of suicide within 3 and 12 months of inpatient care compared with non-ECT. The association was significant in several, but not all, analyses. Furthermore, ECT was associated with reduced all-cause mortality within 3 and 12 months of inpatient care compared with non-ECT.

Our findings are inconsistent with those of a previous study by Jørgensen et al,^8^ which reported that ECT was associated with an increased risk of suicide. This previous study included patients with mild depression, outpatients, and patients with an unspecified severity of depression. These patients seldom receive ECT, and, if they do, they are likely to differ from other patients with mild depression in several ways. For example, they may have responded poorly to previous treatments or be more likely to have psychiatric comorbidities. In fact, in this previous study, the HR for the association between ECT and suicide decreased with an increasing severity of depression, from 6.99 (95% CI, 3.30-14.43) for mild depression to 1.10 (95% CI, 0.55-2.20) for severe depression with psychosis^8^ Thus, in patients with severe depression and psychosis, there was no significant association between ECT and suicide risk, and this finding could have been influenced by insufficient statistical power. Moreover, the indications for ECT may be more liberal in Sweden than in Denmark, which facilitates comparisons by reducing indication bias. Furthermore, the previous study did not control for several risk factors for suicide, such as family history of mental disorders or suicide and comorbidities. Another study by Munk-Olsen et al^9^ reported that ECT was associated with an increased risk of suicide within 1 week after its administration and a marginally increased risk after 4 weeks compared with a control group of patients who had not received ECT. However, that study included several psychiatric diagnoses that were classified into larger diagnosis groups, whereby all unipolar diagnoses were combined into a single diagnosis group regardless of severity. Therefore, the increased risk of suicide found in patients receiving ECT might have been due to bias, because ECT is more commonly used to treat patients with severe depression and suicide risk. Indeed, our results suggest that patients receiving ECT had a more severe form of depression than other inpatients with depression. Furthermore, the study by Munk-Olsen et al^9^ was small and did not adjust for several potential confounding factors, including previous suicide attempts, psychiatric and somatic comorbidities, and a family history of mental disorder or suicide. A previous study with a follow-up period of several years showed that ECT was associated with a decreased risk of suicide.^10^ However, in that study, different inclusion criteria were applied to the ECT and control groups: patients in the non-ECT group were required to have at least 3 previous psychiatric hospitalizations, whereas patients in the ECT group were excluded if they had a history of unipolar or bipolar disorders. The different inclusion criteria introduce a potential bias and mean the results are uncertain. Thus, these findings cannot be considered to reliably support our result that ECT was associated with a decreased risk of suicide.

The association between ECT and a decreased risk of suicide was significantly greater in older age groups than in younger age groups. Older age is a risk factor for suicide, and the distinct association between ECT and a reduced risk of suicide in older adults is therefore important.^12^ This association was in line with our hypothesis, which was based on the positive effect of ECT on response rates in older patients.^5^ There was a significant interaction effect in reduction of the suicide rate between treatment and severity of depression. The greatest benefit of ECT was found for psychotic depression, whereas no significant benefit was noted for moderate depression. Thus, it was unclear whether the association between ECT and a reduced suicide risk is generalizable to outpatients with less severe depression.

### Limitations

The present study has some limitations that should be noted. First, patients who received ECT were more likely to have severe depression and severe depression with psychosis than patients in the non-ECT group. Although we adjusted for these differences in severity by matching and multivariate analysis, some differences may remain. It is possible that the severity of depression was higher in the ECT group even after the adjustment. Second, depression with suicide risk is an indication for ECT, and the risk of suicide judged by the treating psychiatrist was thus probably higher in the ECT group than in the non-ECT group. Therefore, we may have underestimated the association of ECT with reduced suicide risk. Third, we adjusted for many possible confounders but cannot exclude the possibility that additional factors affected the outcome that were not included in the analyses. For example, the main analysis did not account for differences in treatment during the follow-up period because such differences could arise from many factors, including remaining or recurring symptoms. Nevertheless, patients who received ECT may have been managed more intensively during the follow-up period, which could explain some of the observed differences. Indeed, in the sensitivity analysis adjusting for treatment with antidepressants and/or lithium during the follow-up, the difference in suicide risk between the ECT and non-ECT groups was reduced, which may be explained by the beneficial association of lithium with suicide risk and the higher prevalence of lithium treatment in the ECT group. The ECT group also had more inpatient episodes, which was in line with the hypothesis that patients in this group may have been treated more intensively. Fourth, suicide is uncommon even in at-risk cohorts, which limited the number of events, made the CIs wide, and may have contributed to the statistically insignificant results in some analyses. Future studies could use even larger cohorts to increase the precision regarding which subgroups benefit from reduced suicide risk after ECT. This could be achieved by combining data from similar registers in countries such as Sweden and Denmark. Future studies also need to address which patients may benefit from reduced suicide risk with the different prophylactic treatments that are available, including antidepressants, lithium, and continuation ECT.

## Conclusions

In this cohort study, ECT for depression was associated with a reduced risk of suicide within 3 and 12 months compared with non-ECT. This study supports the continued use of ECT to prevent suicide among inpatients with severe depression, especially older patients and patients with psychotic depression. This study was limited by potential residual confounding due to indication and management during the follow-up period.

## Notes

Supplement. **eTable 1.** List of ICD-10 Codes Used **eTable 2.** Anatomical Therapeutic Chemical Classification (ATC) Codes **eTable 3.** National Classification of Health Interventions Codes (KVÅ) for ECT **eTable 4.** Details of ECT **eTable 5.** Baseline Characteristics of Patients With Depression Treated With and Without Electroconvulsive Therapy Based on Inpatient Episodes **eTable 6.** Results of Univariate and Multivariate Cox Regression Analyses of the Risk of Suicide Within 3 Months After Discharge for Patients With Depression, Based on Inpatient Episodes
