# Supplementary material for: Electroconvulsive Therapy and the Risk of Suicide in Hospitalized Patients With Major Depressive Disorder
Source: JAMA Netw Open. 2021 Jul 21;4(7):e2116589. doi: 10.1001/jamanetworkopen.2021.16589 (PMC8295734; doi:10.1001/jamanetworkopen.2021.16589)
Supplement: Supplement. — eTable 1. List of ICD-10 Codes Used eTable 2. Anatomical Therapeutic Chemical Classification (ATC) Codes eTable 3. National Classification of Health Interventions Codes (KVÅ) for ECT eTable 4. Details of ECT eTable 5. Baseline Characteristics of Patients With Depression Treated With and Without Electroconvulsive Therapy Based on Inpatient Episodes eTable 6. Results of Univariate and Multivariate Cox Regression Analyses of the Risk of Suicide Within 3 Months After Discharge for Patients With Depression, Based on Inpatient Episodes [file jamanetwopen-e2116589-s001.pdf]

Supplementary Online Content

Rönnqvist I, Nilsson FK, Nordenskjöld A. Electroconvulsive therapy and the risk of suicide in hospitalized patients with major depressive disorder. *JAMA Netw Open*. 2021;4(7):e2116589. doi:10.1001/jamanetworkopen.2021.16589

eTable 1. List of ICD-10 Codes Used

eTable 2. Anatomical Therapeutic Chemical Classification (ATC) Codes

eTable 3. National Classification of Health Interventions
Codes (KVÅ) for ECT

eTable 4. Details of ECT

eTable 5. Baseline Characteristics of Patients With Depression Treated With and Without Electroconvulsive Therapy Based on Inpatient Episodes

eTable 6. Results of Univariate and Multivariate Cox Regression Analyses of the Risk of Suicide Within 3 Months After Discharge for Patients With Depression, Based on Inpatient Episodes

This supplementary material has been provided by the authors to give readers additional information about their work.

© 2021 Rönnqvist I et al. *JAMA Network Open.*

eTable 1. List of ICD-10 Codes Used

	**ICD-10 codes**
**Main diagnosis**	
Moderate depression	F32.1, F33.1
Severe depression without psychotic symptoms	F32.2, F33.2
Severe depression with psychotic symptoms	F32.3, F33.3
**Psychiatric comorbidity**	
Anxiety disorder	F40-F48
Personality disorder	F60-F61
Alcohol use disorder	F10
Substance use disorder	F11–F16, F18–F19
**Somatic comorbidity**	
Ischemic heart disease	I20–I25
Heart failure	I50
Stroke	I60–I66
Cancer	C00–C97
Spinal disease	M45–M54
**Suicide attempt**	
Poisoning	X60–X69, Y10–Y19
Other method	X70–X84, Y20–Y34
**Suicide**	X60–X84, Y10–Y34

© 2021 Rönnqvist I et al. *JAMA Network Open.*

eTable 2. Anatomical Therapeutic Chemical Classification (ATC) Codes

	**ATC**
Antidepressants	N06A
Lithium	N05AN
Drugs used in diabetes	A10
Drugs for obstructive airway diseases	R03

eTable 3. National Classification of Health Interventions Codes (KVÅ) for ECT

	**KVÅ**
ECT, unspecified	DA006
ECT, unilateral	DA024
ECT, bilateral	DA025

eTable 4. Details of ECT

	Mean (SD)	N
**Pulse width (ms)**	0.50 (0.12)	3818
**Frequency (Hz)**	62 (20)	3801
**Duration (s)**	6.7 (1.4)	3778
**Current (s)**	841 (57)	3784
**Charge (mA)**	346 (147)	3817
**Seizure duration (s)**	49 (23)	3705

© 2021 Rönnqvist I et al. *JAMA Network Open.*

eTable 5. Baseline Characteristics of Patients With Depression Treated With and Without Electroconvulsive Therapy Based on Inpatient Episodes

	**ECT group** (n = 11578)	**Non-ECT group** (n = 31591)	
	n (%)	n (%)	**P-value**
**Sex**			<0.001
Male	4507 (38.9)	13786 (43.6)	
Female	7071 (61.1)	17805 (56.4)	
**Age, mean years (SD)**	57.2 (18.5)	46.3 (19.1)	
**Marital status**			<0.001
Married/cohabiting	4483 (38.7)	8313 (26.3)	
Divorced	2103 (18.2)	5979 (18.9)	
Widowed	1250 (10.8)	1754 (5.6)	
Unmarried or unknown	3742 (32.3)	15545 (49.2)	
**Household**			<0.001
Not living alone	6094 (52.6)	15583 (49.3)	
Living alone or unknown	5484 (47.4)	16008 (50.7)	
**Education level**			<0.001
Low (≤9 years) or unknown	3009 (25.7)	8896 (27.9)	
Middle (9–12 years)	5290 (45.2)	14647 (45.9)	
High (>12 years)	3413 (29.1)	8366 (26.2)	
**Parental education level**			<0.001
Low (≤9 years)	2535 (21.9)	5551 (17.6)	
Middle (9–12 years)	2728 (23.6)	9966 (31.5)	
High (>12 years)	1718 (14.8)	6816 (21.6)	
Unknown	4597 (39.7)	9258 (29.3)	
**Employment status**			<0.001
Employed	5464 (47.2)	17972 (56.9)	
Unemployed or unknown	6114 (52.8)	13619 (43.1)	
**Severity of depression**			<0.001
Moderate (F32.1, F33.1)	2405 (20.8)	15220 (48.2)	
Severe without psychosis (F32.2, F33.2)	6243 (53.9)	11956 (37.8)	
Severe with psychosis (F32.3, F33.3)	2930 (25.3)	4415 (14.0)	
**Psychiatric comorbidity**			
Anxiety disorder	4286 (37.0)	11058 (35.0)	<0.001
Personality disorder	919 (7.9)	2719 (8.6)	0.027
Alcohol use disorder	1027 (8.9)	4214 (13.3)	<0.001
Substance use disorder	1118 (9.7)	3915 (12.4)	<0.001
**Somatic comorbidity**			
Drugs used in diabetes	1071 (9.3)	2447 (7.7)	<0.001
Drugs for obstructive airway diseases	2678 (23.1)	7875 (24.9)	<0.001
Ischemic heart disease	673 (5.8)	1662 (5.3)	0.025
Heart failure	278 (2.4)	852 (2.7)	0.088
Stroke	411 (3.5)	1161 (3.7)	0.538
Cancer	773 (6.7)	1428 (4.5)	<0.001

© 2021 Rönnqvist I et al. *JAMA Network Open.*

Spinal disease	788 (6.8)	2291 (7.3)	0.111
**Suicide attempt – poisoning**			<0.001
Within 365 days	979 (8.5)	3406 (10.8)	
Earlier than 365 days	1041 (9.0)	3133 (9.9)	
No	9558 (82.6)	25052 (79.3)	
**Suicide attempt – other method**			0.288
Within 365 days	232 (2.0)	711 (2.3)	
Later than 365 days	327 (2.8)	875 (2.8)	
No	11019 (95.2)	30005 (95.0)	
**Pharmacotherapy within 3 months before admission**			
Antidepressants	9356 (80.7)	20737 (65.6)	<0.001
Lithium	607 (5.2)	864 (2.7)	<0.001
**Pharmacotherapy within 3 months after admission**			
Antidepressants	10036 (86.7)	26882 (85.1)	<0.001
Lithium	1254 (10.8)	1263 (4.0)	<0.001
**Pharmacotherapy within 12 months after admission**			
Antidepressants	11090 (95.8)	29023 (91.9)	<0.001
Lithium	2101 (18.1)	2132 (6.7)	<0.001
**Compulsory psychiatric treatment**			<0.001
Yes	1929 (16.7)	4464 (14.1)	
No	9649 (83.3)	27127 (85.9)	
**Family history of mental disorder**			0.734
Yes	1304 (11.3)	3595 (11.4)	
No	10274 (88.7)	27996 (88.6)	
**Family history of suicide**			0.024
Yes	453 (3.9)	1092 (3.5)	
No	11125 (96.1)	30499 (96.5)	

ECT: electroconvulsive therapy; SD: standard deviation.

© 2021 Rönnqvist I et al. *JAMA Network Open.*

eTable 6. Results of Univariate and Multivariate Cox Regression Analyses of the Risk of Suicide Within 3 Months After Discharge for Patients With Depression, Based on Inpatient Episodes

	HR (95% CI)	P-value	aHR (95% CI)	P-value
**Treatment**				
Non-ECT	Reference		Reference	
ECT	0.66 (0.47–0.93)	0.017	0.58 (0.40–0.83)	0.003
**Sex**				
Male	Reference		Reference	
Female	0.41 (0.31–0.54)	<0.001	0.46 (0.34–0.61)	<0.001
**Age**	1.01 (1.00–1.01)	0.054	1.03 (1.02–1.04)	<0.001
**Marital status**				
Married/cohabiting	Reference		Reference	
Divorced	0.97 (0.65–1.44)	0.86	1.05 (0.64–1.70)	0.86
Widowed	0.34 (0.14–0.83)	0.02	0.35 (0.13–0.92)	0.03
Unmarried or unknown	1.06 (0.77–1.44)	0.74	1.41 (0.91–2.18)	0.13
**Household**				
Not living alone	Reference		Reference	
Living alone or unknown	1.03 (0.79–1.35)	0.83	0.93 (0.65–1.32)	0.67
**Education level**				
Low (≤9 years) or unknown	Reference		Reference	
Middle (9–12 years)	1.28 (0.90–1.81)	0.17	1.28 (0.89–1.83)	0.18
High (>12 years)	1.46 (0.999–2.12)	0.051	1.42 (0.95–2.11)	0.09
**Parental education level**				
Low	1.28 (0.87–1.88)	0.21	1.21 (0.80–1.82)	0.37
Middle	0.94 (0.65–1.36)	0.75	1.13 (0.72–1.76)	0.60
High	1.43 (0.99–2.06)	0.60	1.82 (1.13–2.91)	0.01
Unknown	Reference		Reference	
**Employment status**				
Employed	1.12 (0.85–1.47)	0.43	1.07 (0.78–1.46)	0.69
Unemployed or unknown	Reference		Reference	
**Severity of depression**				
Moderate (F32.1, F33.1)	Reference		Reference	
Severe without psychosis (F32.2, F33.2)	1.28 (0.95–1.73)	0.11	1.31 (0.96–1.78)	0.09
Severe with psychosis (F32.3, F33.3)	1.16 (0.78–1.72)	0.48	1.13 (0.74–1.74)	0.56
**Psychiatric comorbidity**				
Anxiety disorder	0.86 (0.66–1.18)	0.41	0.99 (0.72–1.37)	0.97
Personality disorder	0.54 (0.29–1.02)	0.058	0.60 (0.30–1.19)	0.15
Alcohol use disorder	1.30 (0.89–1.89)	0.17	1.07 (0.70–1.62)	0.76
Substance use disorder	1.21 (0.82–1.79)	0.34	1.31 (0.84–2.05)	0.23
**Somatic comorbidity**				
Drugs used in diabetes	0.69 (0.38–1.23)	0.21	0.67 (0.37–1.21)	0.18
Drugs for obstructive airway diseases	0.68 (0.48–0.97)	0.033	0.76 (0.53–1.09)	0.13

© 2021 Rönnqvist I et al. *JAMA Network Open.*

Ischemic heart disease	0.97 (0.51–1.79)	0.93	0.83 (0.44–1.59)	0.58
Heart failure	0.93 (0.38–2.25)	0.87	0.81 (0.32–2.05)	0.66
Stroke	0.52 (0.19–1.39)	0.19	0.41 (0.15–1.13)	0.09
Cancer	1.88 (1.17–3.01)	0.009	1.86 (1.14–3.05)	0.01
Spinal disease	0.79 (0.44–1.42)	0.43	0.77 (0.43–1.40)	0.40
**Suicide attempt – poisoning**				
Within 365 days 2	1.44 (0.97–2.12)	0.07	1.66 (1.10–2.52)	0.02
Earlier than 365 days 1	0.75 (0.44–1.28)	0.29	0.93 (0.53–1.63)	0.79
No	Reference		Reference	
**Suicide attempt – other method**				
Within 365 days	2.24 (1.19–4.22)	0.01	1.97 (1.03–3.77)	0.04
Earlier than 365 days	0.87 (0.36–2.12)	0.76	1.01 (0.41–2.53)	0.98
No	Reference		Reference	
**Pharmacotherapy**				
Antidepressants	0.69 (0.48–1.001)	0.051	0.77 (0.52–1.13)	0.18
Lithium	0.64 (0.35–1.18)	0.16	0.71 (0.38–1.32)	0.28
**Compulsory psychiatric treatment**				
Yes	1.23 (0.87–1.76)	0.25	1.19 (0.82–1.72)	0.37
No	Reference		Reference	
**Family history of mental disorder**				
Yes	1.46 (1.01–2.12)	0.046	1.52 (1.04–2.21)	0.03
No	Reference		Reference	
**Family history of suicide**				
Yes	1.35 (0.72–2.55)	0.35	1.19 (0.63–2.25)	0.60
No	Reference		Reference	

aHR: adjusted hazard ratio; ECT: electroconvulsive therapy; HR: hazard ratio.

© 2021 Rönnqvist I et al. *JAMA Network Open.*
